# Trends in the use of complementary and alternative medicine between 1987 and 2021 in Denmark

**DOI:** 10.1186/s12906-023-04327-8

**Published:** 2024-01-06

**Authors:** Sofie Rossen Møller, Ola Ekholm, Anne Illemann Christensen

**Affiliations:** National Institute of Public Health, University of Southern Denmark, Studiestraede 6, Copenhagen K, 1455 Denmark

**Keywords:** Complementary and alternative medicine, Questionnaire survey, Trends, Sociodemographic characterisation

## Abstract

**Background:**

Complementary and alternative medicine (CAM) has been widely and increasingly used worldwide during the past decades. Nevertheless, studies in long-term trends of CAM use are limited. The aim of this study was to assess long-term trends in the prevalence of CAM use (both overall and for specific CAMs) between 1987 and 2021 in the adult Danish population and to examine certain sociodemographic characteristics of CAM users.

**Methods:**

Data derived from nationally representative health surveys in the general adult population (≥ 16 years) in Denmark (the Danish Health and Morbidity Surveys) conducted in 1987, 1994, 2000, 2005, 2010, 2013, 2017, and 2021. The response proportion declined from 79.9% in 1987 to 45.4% in 2021. CAM use was assessed by questions on ever use of specific types of CAMs and overall use within the past 12 months. Differences in use of CAMs across educational levels were assessed using the Slope Index of Inequality (SII).

**Results:**

An overall increase in the prevalence of CAM use within the past 12 months was found between 1987 (10.0%) and 2021 (24.0%). However, a stagnation was observed between 2010 and 2017, after which the prevalence decreased in 2021. In all survey waves, the prevalence was higher among women than men. For both sexes, the prevalence tended to be highest among respondents aged 25–44 years and 45–64 years. The group with 13–14 years of education had the highest prevalence of CAM use compared to the other educational groups (< 10 years, 10–12 years, and ≥ 15 years). SII values for both men and women increased between 1987 and 2021, which indicates an increase in differences of CAM use across educational groups. In all survey waves the most frequently used CAMs included massage and other manipulative therapies, acupuncture, and reflexology.

**Conclusions:**

The use of CAM has increased markedly within the last decades and recently stagnated at high levels, which underlines the importance of securing high quality information and education for the public, health professionals, and legislators to ensure and promote safe use of CAMs.

**Supplementary Information:**

The online version contains supplementary material available at 10.1186/s12906-023-04327-8.

## Background

Complementary and alternative medicine (CAM) has been defined by the World Health Organization as a broad set of health care practices that are not part of a given country’s own traditional or conventional medicine and are not fully integrated into the dominant health care system [1]. During the past decades CAM has been widely and increasingly used in most Western countries [2–6].

In Europe, some of the most common CAMs include herbal medicine, homeopathy, chiropractic treatment, acupuncture, and reflexology [7]. In 2018, it was estimated that 26% of the general population in various European countries had used at least one type of CAM therapy within the past 12 months [6]. However, the demarcation between CAMs and conventional therapies varies over time and across countries, and thus, the prevalence rates of CAM use depend highly on the types of services, products, and self-help practices that are included in the definition of CAM [8, 9].

Data on the prevalence of CAM use is deemed beneficial in order to assess the implications of CAM use for healthcare systems and determine the requirements for research, education and regulation in the CAM field [10].

In Denmark, the use of CAM in the general adult population has been assessed regularly since 1987 through the Danish Health and Morbidity Surveys [11]. This includes assessments in the prevalence of several specific types of CAMs, such as acupuncture, reflexology, and homeopathy. Hence, the data from these surveys possess a unique possibility of assessing the trends in CAM use in Denmark over a period of more than 30 years.

The aim of the present study was to assess the trends in prevalence of CAM use (both overall and for specific CAMs) between 1987 and 2021 in the general adult population in Denmark. Furthermore, we aimed to describe certain characteristics of CAM users, including sex, age, and educational level. Lastly, we aimed to examine educational differences in the trends in CAM use using the Slope Index of Inequality.

## Methods

### Study design and participants

The data used in the present study derived from the Danish Health and Morbidity Surveys, which are national health surveys that have been conducted regularly since 1987 [11]. These surveys assess the status and trends in health and morbidity as well as factors that may influence health status in the general adult population (≥ 16 years) in Denmark. In the present study, we used data from all survey waves, i.e., from 1987, 1994, 2000, 2005, 2010, 2013, 2017, and 2021.

The survey samples were drawn at random from the Danish Civil Registration System in which all citizens with an official residence in Denmark are registered by a unique personal registration number [12]. In 1987, 1994, 2000, and 2005 data were collected via face-to-face interviews at the respondents’ home [13]. In 2010, 2013, 2017, and 2021 data were collected via self-administered questionnaires [11]. A previous study has concluded that the responses in terms of CAM use did not vary depending on the use of either face-to-face interviews or self-administered questionnaires, and thus, the use of different data collection modes did not seem to influence the response patterns for CAM use [14].

The sample designs of the surveys have been described in detail elsewhere [11, 13]. The response proportions were 79.9% in 1987 (5,950 invited), 77.8% in 1994 (5,995 invited), 74.2% in 2000 (22,484 invited), 66.7% in 2005 (21,832 invited), 60.7% in 2010 (25,000 invited), 57.1% in 2013 (25,000 invited), 56.1% in 2017 (25,000 invited), and 45.4% in 2021 (25,000 invited). The surveys in 1987, 1994 and 2000 were carried out in three rounds during the year. In 2005, data were collected continuously from May 2005 to March 2006. The survey samples in the first four waves were drawn among Danish citizens [13]. In 2010, 2013, 2017 and 2021 the surveys were carried out between February and May and the survey samples were drawn among individuals with a permanent residence in Denmark [11].

### Assessment of CAM

In 1987, 1994, and 2000, CAM use was assessed by asking the respondents whether they had ever been treated by practitioners outside the public healthcare system and used any of the listed types of providers or forms of treatment. The response categories included the different types of CAMs that appear from Table 1. Respondents who answered that they had used at least one of the different types of CAMs were furthermore asked whether it had taken place within the past year or if it had happened more than a year ago. Hence, it was not possible to estimate the past year prevalence for each of the specific CAMs in the first three waves. In 2005, 2010, 2013, 2017, and 2021, CAM use was assessed by asking the same question as the previous years. However, the response categories were slightly altered. For each of the listed CAMs the respondents were given the following response categories: ‘Yes, within the past 12 months’, ‘Yes, but previously than within the past 12 months’, and ‘No’. A list of the specific types of CAMs included in the questionnaire in all the survey waves is shown in an additional file [Additional file 1]. It appears from the additional file that the included CAMs has varied slightly across the survey waves. For instance, psychotherapy was only included in the survey wave from 1987.

**Table 1:** Ever use of CAM from 1987–2021 overall and for men and women. Percentages*

	1987	1994	2000	2005	2010	2013	2017	2021
**Reflexology**	9.1	14.9	20.1	20.7	23.3	21.4	20.4	20.2
*Men*	*6.3*	*9.6*	*13.0*	*13.1*	*16.0*	*14.3*	*13.1*	*12.3*
*Women*	*11.7*	*19.9*	*26.9*	*27.9*	*30.5*	*28.4*	*27.2*	*27.7*
**Acupuncture**	2.8	6.7	11.1	16.0	24.0	25.6	28.8	29.0
*Men*	*1.9*	*5.1*	*8.4*	*12.0*	*19.1*	*19.6*	*22.1*	*21,8*
*Women*	*3.7*	*8.3*	*13.7*	*20.0*	*28.9*	*31.4*	*35.0*	*35.9*
**Faith healing and/or clairvoyance**	0.9	2.5	4.4	6.0	8.2	8.2	8.6	8.7
*Men*	*0.5*	*1.7*	*2.6*	*3.5*	*4.7*	*4.6*	*4.6*	*3.9*
*Women*	*1.3*	*3.4*	*6.1*	*8.5*	*11.6*	*11.8*	*12.3*	*13.2*
**Homeopathy**	-	-	-	3.5	5.5	5.9	5.6	5.4
*Men*				*2.1*	*2.8*	*3.1*	*3.0*	*2.5*
*Women*				*4.9*	*8.2*	*8.6*	*7,9*	*8.2*
**Nutritional therapy**	-	-	-	2.4	6.8	6.4	6.3	6.7
*Men*				*1.4*	*4.4*	*3.9*	*3.9*	*4.1*
*Women*				*3.5*	*9.2*	*8.7*	*8.6*	*9.1*
**Massage and other manipulative ** **therapies**	5.3	9.2	15.5	21.5	34.3	34.7	37.0	41.2
*Men*	*4.9*	*7.9*	*12.6*	*17.5*	*28.8*	*28,5*	*31.4*	*33.9*
*Women*	*5.6*	*10.4*	*18.2*	*25.2*	*39.7*	*40,8*	*42,4*	*48.1*
**Craniosacral therapy**	-	-	-	3.1	6.1	6.7	7.7	8.6
*Men*				*1.6*	*2.8*	*3,4*	*3.9*	*4.0*
*Women*				*4.6*	*9.4*	*9,8*	*11.2*	*12.9*
**Biopathy, naturopathy**	-	-	-	1.0	1.7	2.0	1.6	1.5
*Men*				*0.4*	*0.8*	*1.2*	*0.7*	*0.7*
*Women*				*1.5*	*2.6*	*2.7*	*2.3*	*2.3*
**Kinesiology**	-	-	-	3.5	4.2	4.4	4.1	4.0
*Men*				*1.8*	*2.3*	*2.3*	*2.4*	*2.0*
*Women*				*5.1*	*6.1*	*6.4*	*5.8*	*6.0*
**Phytoterapi**	-	-	-	-	2.0	2.3	2.3	1.9
*Men*					*1.3*	*1.7*	*1.7*	*1.2*
*Women*					*2.6*	*2.9*	*2.8*	*2.6*
**Other**	2.5	3.1	4.7	2.2	3.2	3.7	3.6	3.3
*Men*	*2.9*	*2.9*	*4.2*	*1.5*	*1.6*	*2.0*	*1.8*	*1.7*
*Women*	*2.0*	*3.3*	*5.0*	*2.9*	*4.7*	*5.4*	*5.3*	*4.8*
**Total**	23.2	33.1	43.7	44.4	52.8	53.2	54.8	56.2

*Note that the total prevalence of CAM use in 1987–2005 includes therapies not shown in the table.

### Demographic variables

Information on sex, age, and civil status was obtained from the sampling frame (Danish Civil Registration System) [12]. Information on highest completed level of education was self-reported and categorized into < 10 years, 10–12 years, 13–14 years and ≥ 15 years.

### Statistical analyses

Descriptive statistics of all the categorical variables are presented with frequencies (n) and percentages (%). Χ^2^-tests were used to evaluate differences in study population characteristics. Age-adjusted percentages of CAM use within the past 12 months by educational level were calculated using the adult Danish population in 2021 as the standard population.

Due to a general increase in educational levels in Denmark during the study period, an additional analysis of differences in use of CAM across educational levels was carried out using the Slope Index of Inequality (SII). SII is an absolute measure of the linear association between education and the prevalence of a given indicator [15]. We conducted a linear regression of the prevalence of CAM use on ridit scores of the prevalence of each educational group while controlling for age. Analyses involving educational level were restricted to individuals aged 25 years or older.

Calibrated weights were constructed by Statistics Denmark and applied to the data to reduce the impact of non-response in each wave separately. The weights were based on the following information from administrative registers: sex, age, municipality of residence, highest completed level of education, income, marital status, ethnic background, number of visits to the general practitioner and hospitalizations within a year for each survey wave, occupational status and home owner/tenant status [11]. In the analyses, those that were less likely to participate were given a higher weight to represent the larger number of non-respondents with similar characteristics. Likewise, those more likely to participate were given a lower weight. All statistical procedures were conducted using SAS version 9.4.

## Results

The baseline characteristics of the respondents from 1987 to 2021 are presented in Table 2. During this period, the proportion of men has decreased from 48.8 to 43.9%. The proportion of respondents aged between 16 and 44 years has decreased, while the proportion of respondents aged 45 years or older has increased substantially. Likewise, the proportion of respondents with less than 13 years of education has decreased, while the proportion with more than 15 years of education has increased considerably.

**Table 2:** Basic characteristics of the study populations. Percentages

	1987 *n = 4,752*	1994 *n = 4,667*	2000 *n = 16,688*	2005 *n = 14,566*	2010 *n = 15,165*	2013 *n = 14,265*	2017 *n = 14,022*	2021 *n = 11,346*
**Sex** (*p* < 0.0001)								
Men	48.8	47.9	49.1	48.6	45.9	45.2	45.8	43.9
Women	51.2	52.1	51.0	51.4	54.1	54.8	54.2	56.1
**Age** (*p* < 0.0001)								
16–24 years	18.1	15.9	13.1	9.8	11.0	12.1	11.6	10.1
25–44 years	37.6	38.2	34.9	33.2	27.7	24.3	25.0	20.2
45–64 years	26.2	28.3	34.0	36.3	37.9	37.0	36.2	34.7
65–74 years	10.3	10.6	9.7	12.1	14.7	17.0	17.4	19.8
≥75 years	7.9	7.2	8.4	8.6	8.7	9.6	9.9	15.2
**Education** (*p* < 0.0001)								
<10 years	32.8	23.8	19.8	15.9	12.7	11.8	8.9	8.1
10–12 years	30.5	29.9	30.5	28.4	24.8	24.1	22.1	21.4
13–14 years	25.2	27.6	29.3	31.3	27.9	28.2	29.9	29.1
≥15 years	7.3	14.3	18.5	22.1	29.4	31.7	35.1	38.5
Other	4.1	4.4	1.9	2.4	5.3	4.2	3.9	3.0
**Cohabitation status** (*p* < 0.0001)								
Married	53.0	50.4	52.5	55.1	59.5	57.3	53.1	53.2
Cohabiting	3.2	16.7	15.6	15.5	11.9	12.3	14.0	13.4
Single, separated or divorced	4.8	5.6	5.8	6.2	6.6	6.0	7.5	8.3
Widowed	7.8	7.4	7.6	6.9	5.9	6.1	5.6	6.7
Single, unmarried	31.2	20.0	18.5	16.2	16.1	18.4	19.9	18.4

**Fig. 1:**
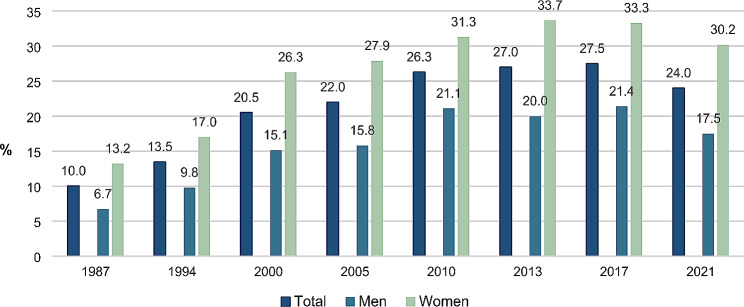
Prevalence of CAM use within the past 12 months in total and by sex. Percentages

Figure 1 shows that overall, there has been a substantial increase in the prevalence of CAM use within the past 12 months from 1987 (10.0%) to 2021 (24.0%). From 1987 to 2010, the prevalence increased gradually, after which the increase stagnated in 2013 and then decreased between 2017 and 2021. In all survey waves, the prevalence has been significantly higher among women compared to men. However, the trend for both men and women has been fairly similar.

**Fig. 2:**
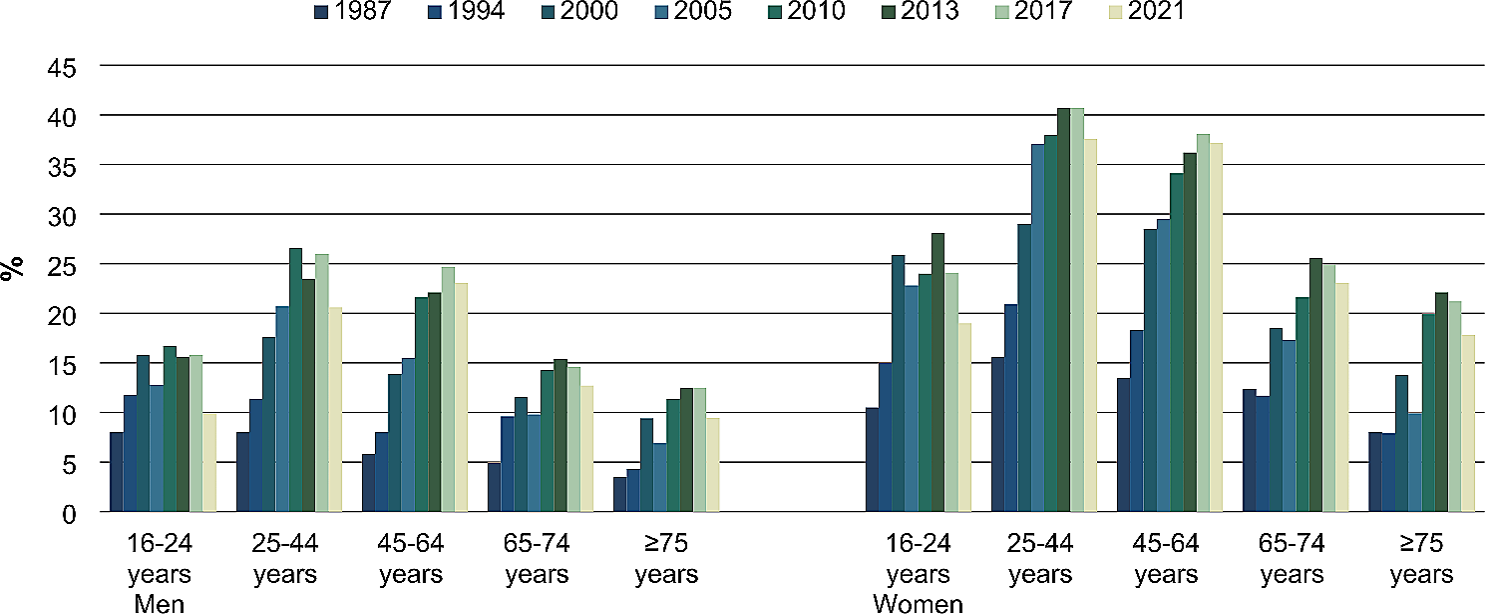
CAM use within the past 12 months by sex and age groups. Percentages

Figure 2 shows an overall increase from 1987 to 2021 in the use of CAM within the past 12 months in all age groups among both men and women. In all waves, the prevalence was higher among women than men in all age groups. Furthermore, the prevalence tended to be highest among the respondents in the age groups from 25 to 44 years and 45–64 years across the years.

**Fig. 3:**
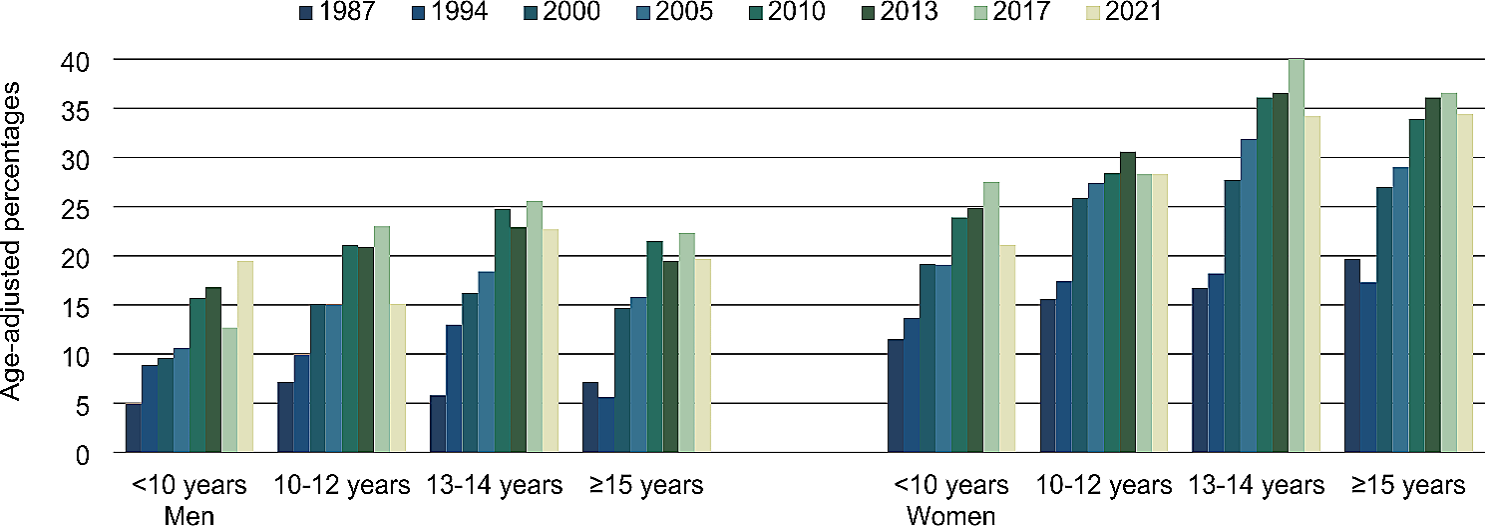
CAM use within the past 12 months by sex and level of education. Age-adjusted percentages

Figure 3 shows an overall increase in the use of CAM within the past 12 months in all educational groups from 1987 to 2021 among both men and women. In most waves, the highest (age-adjusted) proportion was seen in the groups with 13–14 years and ≥ 15 years of education. This tendency was most pronounced among women.

**Fig. 4:**
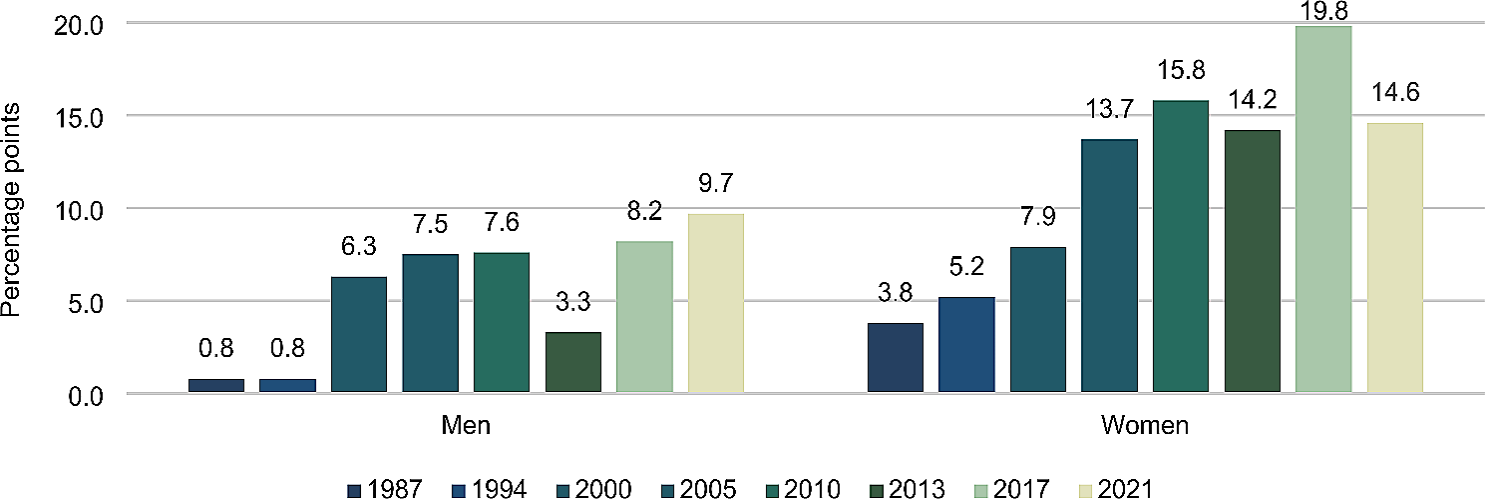
Educational differences in CAM use within the past 12 months using SII values. Percentage points

Figure 4 shows differences in the prevalence of CAM use across the assessed educational groups using SII values. The figure shows an overall increase in the SII values for both men and women between 1987 and 2021. The main increases were observed between 1987 and 2005. In 2021, the SII value among men were 9.7 and 14.6 among women, which means that the difference in the prevalence of CAM use between the highest educational group and the lowest educational group was 9.7 and 14.6 percentage points, respectively.

Table 1 shows that the prevalence of ever use of CAM has increased substantially from 1987 (23.2%) to 2021 (56.2%). Likewise, the prevalence of most of the specific CAMs has increased considerably as well. For instance, the prevalence of the use of acupuncture has increased from 2.8% in 1987 to 29.0% in 2021. In all waves, the three most frequently used CAMs were reflexology, massage and other manipulative therapies, and acupuncture. For all the specific CAMs included in this study, the prevalence of use was higher among women than men throughout the entire period.

## Discussion

Results from this study showed a substantial increase in the prevalence of CAM use within the past 12 months between 1987 (10.0%) and 2010 (26.3%), after which it stagnated. In 2021, the prevalence decreased to 24.0%. The data collection in 2021 was carried out while Denmark had several official COVID-19 restrictions in place, including restricted accessibility to some CAM providers. This was also the case in periods of 2020. Hence, these COVID-19 restrictions are most likely to have affected the use of CAM and may explain the noticeable drop in the prevalence in 2021. In concordance with this, a Norwegian study found that, while the use of some dietary supplementation increased, the use of self-help practices and CAM received from providers decreased during the first wave of the COVID-19 pandemic [16].

In all waves, the prevalence was found to be higher among women than men in all age groups. The prevalence tended to be highest among respondents aged 25–44 years and 45–64 years among both women and men. Regarding educational level, the group with 13–14 years of education had the highest prevalence of CAM use compared to the other educational groups. Ever use of CAM increased considerably between 1987 (23.2%) and 2021 (56.2%). Similarly, the prevalence of ever use of most of the specific CAMs increased considerably as well.

To our knowledge, only a few studies have assessed trends in CAM use over a longer period. Overall, direct comparisons of the results from the present study with those from other studies are difficult because of heterogeneous methodology and demographics of the study populations. However, some general patterns can still be highlighted here. For instance, Canizares and colleagues assessed the prevalence of provider-based CAMs within the past 12 months in Canada in 1994/1995 and 2010/2011 using longitudinal data from a large national population survey [5]. Canizares and colleagues found a prevalence of 14.6% of overall provider-based CAM use in 1994/1995, which had increased to 24.5% in 2010/2011. Apart from a few discrepancies, there was a substantial overlap in the included types of CAMs in their study and our study.

Furthermore, Eisenberg and colleagues investigated trends in CAM use in the United States between 1990 and 1997 [2]. The findings from the surveys showed that 33.8% of the adult US population had used at least one CAM therapy within the past 12 months in 1990, whereas this proportion increased significantly to 42.1% in 1997.

Using data from the study of Eisenberg and colleagues and the National Health Interview Survey (NHIS) from 2002, Tindle and colleagues estimated and compared the prevalence of CAM use within the past 12 months among US adults for specific types of CAMs that were measured similarly in both surveys [17]. They found a prevalence of 36.5% in 1997 and 35.0% in 2002 and, thus, concluded that the prevalence of CAM use had remained stable between 1997 and 2002.

Clarke and her colleagues also assessed the trends in use of CAM in the US adult population from 2002 to 2012. The study concluded that the prevalence of CAM therapy within the past 12 months was rather stable in this period (32.3%, 35.5%, 33.2% in 2002, 2007 and 2012, respectively) [18].

Although, direct comparisons across the different US studies are not feasible due to differences in the included types of CAMs, the studies still provide an overall idea of the development in use of CAM in the US. While a substantial increase in the prevalence of CAM use was found between 1990 and 1997, the prevalence appears to have remained stable between 1997 and 2012. In accordance with Eisenberg et al., we found a substantial increase in the prevalence of CAM use between 1987 and 2000. However, unlike the findings of Tindle et al. and Clarke et al., we found a continued increase in the prevalence until 2010. Moreover, the prevalence estimates found in the US were generally higher than the ones we found among the Danish population throughout the years. Yet, the types of CAMs included in the American studies and our study differed considerably. For instance, unlike our study, the American studies included nonvitamin, nonmineral dietary supplements and relaxation techniques like yoga, tai chi, and qi gong, which were both found to be frequently used in the US [18]. Furthermore, they included chiropractic treatment, which is provided by authorised health care practitioners in Denmark and therefore not considered a CAM therapy in our study.

In general, cross-country comparisons in CAM use are challenging due to variations in what types of therapies that are considered alternative, complementary, and conventional, respectively. However, the Scandinavian countries are fairly similar in terms of overall living conditions, public healthcare systems, patterns of disease and illness, and therapeutic traditions, which enhance the conditions for comparisons [9].

In 2022, a national survey among residents aged 18 years and over was carried out in Norway [19]. In the survey, 38.3% reported having used CAM therapy from a provider, herbs/natural remedies and/or self-help techniques, for health-related purposes. Meanwhile, 24.9% reported that they had received one or more CAM therapies from a provider, inside or outside the public healthcare system. However, these prevalences are not directly comparable to our findings since they were restricted to use of CAMs for health-related purposes. Furthermore, use of CAMs within the public healthcare system was included, whereas our study only included CAMs outside the public healthcare system. Also, other types of CAMs such as herbs and natural remedies (i.e., ginseng, garlic, and ginger) and self-help techniques, including yoga, mindfulness, and meditation were included in the Norwegian study. The study also examined trends in use of CAM every second year between 2012 and 2022. The total use of CAM decreased from 45.3% in 2012 to 35.8% in 2016 after which the use increased to 39.3% in 2020 and remained stable in 2022 (38.3%). The trend in Norway thus differs slightly from our findings of a stagnation in CAM use between 2010 and 2017 and a decrease in 2021. However, as previously stated, these findings are not directly comparable to our findings due to differences in operational definitions.

A study based on national health surveys conducted among Icelandic adults aged 18–75 years found that the prevalence of CAM use over the past 12 months increased from 31.8% in 2006 to 40.2% in 2015 [20]. The increasing trend in Iceland during this period is thus fairly similar to our findings of an increase from 22.0% in 2005 to 27.0% in 2013. However, the Icelandic study only assessed visits to providers of CAM treatments or services, while our question regarding CAM use intended to cover CAMs overall. Furthermore, the Icelandic study included CAM treatments offered by all practitioners in Iceland, whether or not the practitioners operated within or outside the public healthcare system.

Using data from the 2014 European Social Survey (ESS), Kemppainen et al. assessed nationally representative prevalences of CAM use within the past 12 months in 21 European countries [6]. The types of CAMs included in the study were acupuncture, acupressure, Chinese medicine, chiropractic treatment, osteopathy, homeopathy, herbal treatment, hypno-therapy, massage therapy, reflexology, and spiritual healing. In Denmark, the prevalence of CAM use was found to be 32.1%. Despite some disparities in the included types of CAMs, the prevalence found by Kemppainen et al. in Denmark in 2014, was close to our findings of 26.0% in 2013. Kemppainen et al. found prevalences of 28.8% and 31.5% in Norway and Sweden, respectively, which indicates that the prevalences in the Scandinavian countries are similar when measured comparably.

Both regarding CAM use within the past 12 months and ever use of CAM, we found a higher prevalence among women compared to men. This corresponds well with the findings of previous research, which have persistently shown that women use CAM to a greater extent than men [2, 5–7, 10, 20–23]. It has been suggested that a higher use of CAM among women is well in line with previous findings that women generally use more health services than men and that women tend to be more active in their own health promotion and concerned about health issues [21]. Accordingly, this might also make women more likely to actively seek out alternative treatment options such as different CAMs.

In terms of age, we found the highest prevalences of CAM use within the past 12 months among both men and women in the age of 25–44 years and 45–64 years. This is in line with previous findings of several studies that have similarly found the highest prevalences among younger and middle-aged people [2, 6, 9, 10, 22–24]. For instance, Eisenberg et al. found that people aged 35–49 years reported higher rates of CAM use than people at younger and older ages [2]. Hanssen et al. likewise found the highest proportion of respondents reporting ever use of CAM to be within the age group 30–59 years compared to the age groups of < 30 years and ≥ 60 years, respectively, in Norway in 1997 and Stockholm County in 2000 [9]. Similarly, a Swedish study from 2017 found a higher prevalence of CAM use among the age groups of 18–39 years and 40–64 years of age compared to the age group of 65–79 years of age [23].

However, unlike these findings, Kristoffersen et al. found the highest proportion of CAM use within the past 12 months to be among respondents aged 16–29 years, compared to respondents aged 30–59 years and 60 years or more in Norway in 2019 [8].

In our study, the highest prevalences of CAM use were found in the groups with 13–14 years and ≥ 15 years of education. This is in accordance with the findings of several other studies that has equally found higher prevalence’s among those with higher levels of education [2, 5, 6, 8, 9, 20, 22–24]. For instance, Hanssen and colleagues, found a higher prevalence of CAM use among respondents with a higher educational level [9]. Likewise, Kristoffersen et al. found the highest prevalence of CAM use among respondents with a lower university education, followed by respondents with a higher university education, while the lowest prevalence was found among respondents with primary school and secondary school as the highest educational level [8]. Kemppainen and colleagues also found that CAM use was more common among those with a higher education [6]. It has been proposed that individuals with higher education can be expected to be well informed about both CAM and conventional healthcare services and therefore be more likely seek out different solutions to their health problems as well as “second opinions” [9].

During the study period, the proportion with a high level of education has increased in the Danish population [25]. In order to take this into account, an additional analysis of differences in use of CAM across educational levels were carried out using SII. The analysis showed an overall increase in the SII values for both men and women between 1987 and 2021, which means that the difference in the prevalence of CAM use between the highest educational group and the lowest educational group has increased.

It is not possible to derive the causes of the observed trends in CAM use based on the present study. However, it has previously been suggested that patients have become more interested and informed about CAMs due to an increased availability of information on the internet, an increased contact with other cultures that traditionally use CAMs, a distrust of and frustration with the healthcare system, and a growing recognition that many factors contribute to health and well-being [26]. It is possible that some of these factors play a role in the increasing use of CAMs in Denmark shown in the present study between 1987 and 2010. However, the results of the present study also show that the prevalence of CAM use has stagnated at high levels in recent years. The reasons for this stagnation are yet unknown but logically the prevalence would not be expected to increase indefinitely.

The results of the present study indicate that people living in Denmark are seeking out CAMs to a great extent, which stresses the importance of promoting safe use of CAMs in Denmark. This could include research in effects and possible side effects of use of specific CAMs as well as distribution of this information to the general public and healthcare professionals in order to promote informed decisions regarding use of CAM. It could also be useful for healthcare professionals to be aware of the high prevalence of CAM use and gain appropriate knowledge on effects and side effects as well as implications for other concurrent treatments in order to initiate talks with patients about these aspects and secure proper guidance.

The findings of this study must be seen in the light of its strengths and limitations. One of the main strengths of this study is that CAM use was measured rather frequently for a period of more than 30 years in national representative surveys. Furthermore, specific CAM treatments were listed in the questionnaire and CAM use was measured predominantly similar across the different survey waves, which allows for comparisons of the prevalences across all the measured years. To our knowledge, no previous studies have investigated national trends in the overall use of CAM or specific CAMs for a time period that long.

Another strength of the study is the large number of respondents (4,667 to 16,688 respondents) and fairly high response rates (45.4–79.9%) throughout the different survey waves. Still, decreasing response proportions are a major concern. However, calibrated weights were applied to the data to reduce the impact of non-response, and thus, increase representativeness of the target population and generalisability of our findings.

A possible limitation of this study is that the measures of CAM use relied solely on self-reported data. Data on hospitalizations and data on primary care use can often be obtained from administrative registers. However, data on CAM use cannot be obtained from such registers. Hence, information on CAM use is typically obtained from surveys [7, 27]. The accuracy of the data is thus dependent of the respondents’ willingness and abilities to answer correctly, for example in terms of differences in interpretation of the questions and recall. Furthermore, some of the listed CAMs may be interpreted differently due to vague demarcations. For instance, respondents may interpret and thus report *nutritional therapy* differently depending on their understanding of what is included in that type of therapy. In terms of recall, respondents may not remember their 12 months use and ever use of CAM perfectly. Likewise, self-reported data on the use of CAM over time has previously been found to reflect high levels of inconsistencies [28]. Nevertheless, the listing of CAMs in the questionnaire may aid respondent recall.

## Conclusions

Within the past decades, a substantial increase in the use of CAM with signs of stagnation at high levels in recent years has been observed. In all survey waves, the prevalence was higher among women than men in all age groups. The prevalence tended to be highest among respondents aged 25–44 years and 45–64 years and respondents with a higher educational level. The most frequently used CAMs included massage and other manipulative therapies, acupuncture, and reflexology. The continued high prevalence of CAM use in Denmark underlines the importance of promoting safe use of CAMs by securing high quality information and education for the public, health professionals, and legislators.

### Electronic supplementary material

Below is the link to the electronic supplementary material.

Supplementary Material 1

## Data Availability

The dataset supporting the conclusions of this article can be made available from the corresponding author on reasonable request. More information is available at the following web page: https://www.danskernessundhed.dk/Dataudlevering.html.

## Abbreviation

CAM: Complementary and alternative medicine
