# Supplementary material for: Trends in the use of complementary and alternative medicine between 1987 and 2021 in Denmark
Source: BMC Complement Med Ther. 2024 Jan 6;24:23. doi: 10.1186/s12906-023-04327-8 (PMC10770893; doi:10.1186/s12906-023-04327-8)
Supplement: Supplementary file 1 — Supplementary Material 1 [file 12906_2023_4327_MOESM1_ESM.docx]

**Additional file 1**

**Table S1.** Specific types of CAMs included in the questionnaires in the different survey waves

|  | **1987** | **1994** | **2000** | **2005** | **2010** | **2013** | **2017** | **2021** |
| --- | --- | --- | --- | --- | --- | --- | --- | --- |
| Reflexology | X | X | X | X | X | X | X | X |
| Acupuncture | X | X | X | X | X | X | X | X |
| Faith healing and/or clairvoyance | X | X | X | X | X | X | X | X |
| Homeopathy |  |  |  | X | X | X | X | X |
| Natural medicine,  e.g., homeopathy | X | X | X |  |  |  |  |  |
| Nutritional therapy |  |  |  | X | X | X | X | X |
| Dietary advice | X | X | X |  |  |  |  |  |
| Massage and other manipulative  therapies | X | X | X | X | X | X | X | X |
| Craniosacral therapy |  |  |  | X | X | X | X | X |
| Biopathy, naturopathy |  |  |  | X | X | X | X | X |
| Kinesiology |  |  |  | X | X | X | X | X |
| Phytoterapi |  |  |  |  | X | X | X | X |
| Relaxation techniques | X | X |  |  |  |  |  |  |
| Laying on of hands | X | X | X |  |  |  |  |  |
| Use of apparatus,  e.g., mesmeric passes or radionics | X | X | X |  |  |  |  |  |
| Psychotherapy | X |  |  |  |  |  |  |  |
| Hypnosis | X | X | X |  |  |  |  |  |
| Other | X | X | X | X | X | X | X | X |
